# Characteristics of patients positive for COVID-19 and multidrug-resistant organisms in Tennessee, 2020–2021

**DOI:** 10.1017/ash.2022.209

**Published:** 2022-05-16

**Authors:** Carolyn Stover, Erin Hitchingham, Kristina McClanahan, Zoe Durand, Rany Octaria, Christopher Wilson, Allison Chan

## Abstract

**Background:** Multidrug-resistant organisms (MDROs) are a global threat. To track and contain the spread, the Tennessee Department of Health (TDH) performs targeted surveillance of carbapenemase-producing and pan-nonsusceptible organisms. When these MDROs are identified, TDH conducts a containment response and collects epidemiological data, which includes risk factors such as indwelling devices and previous hospitalizations. The impact of the COVID-19 pandemic on these MDROs is not well understood. Therefore, we have described the characteristics of cases positive for both COVID-19 and select MDROs. **Methods:** MDRO investigation data from January 1, 2020–September 30, 2021 were matched with all COVID-19 case data from the TDH statewide surveillance system, National Electronic Disease Surveillance System Base System. MDRO-positive date was defined as the specimen collection date; COVID-19 case date was first defined as the date of symptom onset and if missing, then diagnosis date, and investigation creation date, respectively. Descriptive statistics and Fisher exact tests were calculated using SAS version 9.4 software. **Results:** Among 336 MDRO cases, 50 had a reported SARS-CoV-2–positive result. MDRO types were Enterobacterales (CRE) (n = 31), *Acinetobacter* spp (CRA) (n = 18), and *Pseudomonas aeruginosa* (n = 1). Of these 50 cases, 20 were MDRO-positive before and 30 days after the COVID-19 case date, respectively. Of the 18 CRA cases, 16 (89%), were positive after the COVID-19 case date, compared to 13 (42%) among 31 CRE cases (*P* < .01). Also, 35 patients (70%) had a record of hospitalization, and 22 (63%) had their MDRO specimen collected after the COVID-19 case date (*P* = .37). Of these 22 patients, 4 had their MDRO specimen collected during their COVID-19 hospitalization, with an average duration from admission to MDRO collection date of 17 days (range, 4–36). Among the 50 coinfected cases, 8 died, 7 (88%) of whom were MDRO-positive after their COVID-19 case date. Data on indwelling devices at time of MDRO positivity were completed for 17 cases; 14 had an indwelling device and, among these, 13 (93%) were MDRO-positive after their COVID-19 case date. **Conclusions:** MDRO cases with specimen collections after COVID-19 comprised the majority of hospitalized patients, patients who died, and patients with indwelling devices compared to those with MDROs collected before their COVID-19 case date. These results show a stark difference with CRA as the most common MDRO among post–COVID-19 cases. Our data were limited by reporting gaps. We recognize that patients can remain colonized with MDROs for lengthy durations, which could have result in undetected MDRO cases prior to the COVID-19 case date. More data and analyses are needed to make targeted public health recommendations. However, these findings highlight the burden of MDROs among COVID-19 cases. including adverse health outcomes.

**Funding:** None

**Disclosures:** None

